# Using actor-partner interdependence modeling to understand HPV vaccine acceptance

**DOI:** 10.1371/journal.pone.0181662

**Published:** 2017-07-27

**Authors:** Laura E. VanderDrift, Peter A. Vanable, Katherine E. Bonafide, Jennifer L. Brown, Rebecca A. Bostwick, Michael P. Carey

**Affiliations:** 1 Department of Psychology, Syracuse University, Syracuse, NY, United States of America; 2 Department of Psychiatry and Behavioral Neuroscience, University of Cincinnati, Cincinnati, OH, United States of America; 3 Centers for Behavioral and Preventive Medicine, The Miriam Hospital, Providence, RI, United States of America; 4 Behavioral and Social Sciences, Public Health, Brown University, Providence, RI, United States of America; 5 Psychiatry and Human Behavior, Medicine, Brown University, Providence, RI, United States of America; Universidade Estadual de Maringa, BRAZIL

## Abstract

A wealth of scientific literature has been devoted to understanding what factors influence parents and their adolescent children to obtain the HPV vaccine. This literature is relatively uniform in its methodological approach of sampling individuals (i.e., either parents or adolescents) and examining the predictors of uptake for that individual. To improve understanding of HPV vaccination uptake, we sampled low-income, African American parent-child dyads with either a female (*n* = 93) or a male (*n* = 116) adolescent who had not been vaccinated. Both parents and children completed self-report measures that tapped intent to receive the vaccine and hypothesized predictors of intent (i.e., self-efficacy, beliefs about the vaccine, beliefs about HPV, knowledge of HPV). Using a dyadic analytic approach (i.e., the Actor-Partner Interdependence Model or APIM) [1], we found that parents and their adolescents have different structures of beliefs regarding HPV vaccination (i.e., they are empirically distinguishable). Consistent with prior research, the majority of predictors of an individual’s own intention to vaccinate were individual-level variables; uniquely though, some predictors endorsed by one member of the dyad influenced the intentions held by the other member. Specifically, parents’ reports of HPV severity and their self-efficacy were both associated with adolescents’ intent to obtain the vaccine. Further, adolescents’ beliefs that the vaccine will lead to greater promiscuity or be stigmatizing were associated with parents holding an increased intent to vaccinate. Use APIM improves understanding of HPV vaccination uptake and can be used to guide intervention efforts.

## Introduction

A vaccine exists that combats the most prevalent sexually transmitted infection in the world (i.e., the human papillomavirus, or HPV) and dramatically reduces the incidence of some types of cancer. Yet, as of 2014, roughly 37% of age-eligible adolescents have not completed the vaccine protocol [2], despite its having been approved by the US Food and Drug Administration for 2006 for girls and since 2008 for boys. Uptake rates had increased each year after licensure, providing hope that soon vaccination rates for HPV would be on par with those for other vaccines (e.g., 88% of American adolescents complete a course of the vaccine for tetanus-diphtheria-acellular pertussis) [2]. However, in 2012, the US experienced the first year in which rates of HPV vaccination did not increase for female adolescents [3], and rates of increase since have been more modest than in earlier years [4]. This is disappointing, given the amount of scientific literature dedicated to understanding and increasing HPV vaccination acceptance, and suggests that a fresh approach is needed to understand vaccine acceptance.

Several meta-analyses and systematic reviews of the empirical literature have identified the determinants of vaccination uptake. The strongest predictors of HPV vaccination are perceived susceptibility to HPV, perceived barriers to vaccination (i.e., cost and safety), subjective norms (i.e., social pressure to obtain the vaccine), self-efficacy (i.e., the belief that one can obtain the vaccine), and intentions [4, 5]. All of the meta-analyses and the studies integrated in them have taken an individual perspective on HPV vaccination. Although valuable, an exclusively individual approach is incomplete. In the current work, we examine the dyadic context of this decision, to determine whether a dyadic context can provide additional information to help bolster HPV vaccination uptake.

### Dyadic perspectives on HPV vaccination

Few quantitative examinations of parent and adolescent influence regarding HPV vaccination exist. A November 2015 search of Web of Science using the search terms “dyad*” and “HPV vaccin*” resulted in only three quantitative analyses of dyadic data, and none of these included both male and female adolescents. This is surprising in light of the fact that many individuals begin contributing to their health decisions during adolescence [6], and many parents believe vaccination decisions should be made jointly between parents and their adolescents [7]. When making decisions regarding the HPV vaccination, many parents report that their daughters were involved a moderate to high amount in the decision-making process [8], and the majority of parents report having spoken with their female adolescents about HPV vaccination [9]. Moreover, parents and male adolescents correctly predict what the other prefers with regard to HPV vaccination [10].

Dyadic perspectives in HPV vaccination are important because parents and adolescents often think differently about HPV vaccination. Parents report greater positivity for the vaccination than do their adolescents [10], despite female adolescents believing HPV is more severe and that they are more susceptible to it than their parents believe [11]. When disagreements occur, parents remain the primary decision maker with regard to HPV vaccination. Most parents, and a sizable minority of adolescents, do not feel comfortable with the adolescent making her or his vaccination decision [12]. Even still, adolescents likely influence their parents, even when their parents make the final decision. A qualitative examination found that male adolescents feel that the first health decision they provided input in was the decision their parent made with regard to the HPV vaccine [13].

Of the three dyadic examinations of HPV vaccination, only one utilized a method of analysis that is appropriate for dyadic data [11]. Others used statistics appropriate for individual level data, configured to apply to dyads [10, 14] whereas others simply assessed predictors of self-reported influence [8, 12], or content analyzed qualitative data [13]. The need for a full dyadic analysis of parents and adolescents’ HPV vaccine decision-making is twofold. First, it will explain whether and how parents and adolescents influence each other, which is important for designing interventions for greater vaccine uptake. Second, it will inform whether the individual-level approaches to this topic are theoretically reasonable, or if the final decision is so dyadically-situated that results from individual-level examinations are incomplete.

### Actor-Partner Interdependence Models

**Fig 1:**
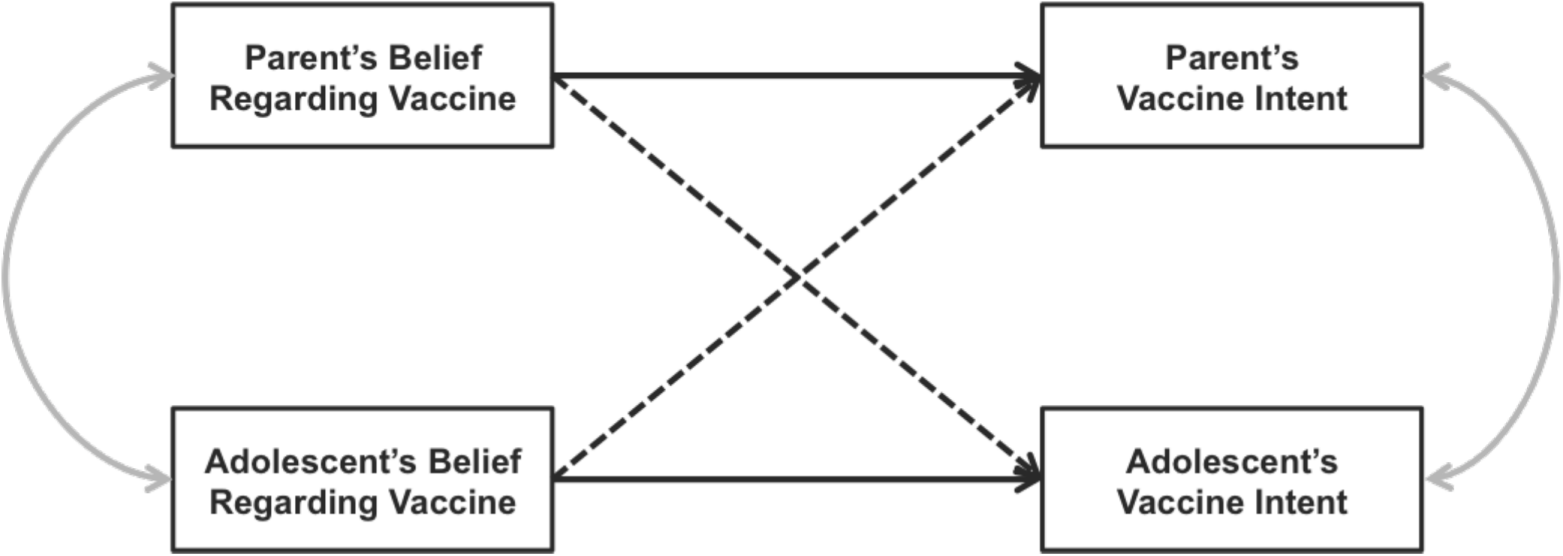
Conceptual diagram of the Actor-Partner Interdependence Model. Solid black lines represent Actor Effects, dotted lines represent Partner Effects, and curved lines represent covariances.

Across myriad domains there are examples of situations in which two actors are interdependent, and thus able to influence each other’s thoughts, emotions, and behavior. In the current work, we use the Actor-Partner Interdependence Model (APIM) to assess HPV vaccination acceptance among parents and their adolescent sons and daughters. APIM is a statistical model that allows for the examination of dyadic processes that models the unique associations within- and between-individuals while accounting for covariance in both the predictors and outcomes [1]. Fig 1 provides a conceptual depiction of the APIM model.

This model is appropriate for understanding HPV vaccination as it is expected that the parents and adolescents will, in large part, share similarities regarding the predictors and outcomes [11], so this covariance must be modeled. Of interest, above and beyond the covariance, APIM will identify whether any of the known within-person predictors of HPV vaccination acceptance produce between-person effects.

Within the APIM, researchers first determine whether dyad members are empirically distinguishable. Whereas parent-adolescent dyads are theoretically distinguishable on the basis of role (i.e., parent or adolescent), empirical distinguishability would hold that they also differ in means, intercepts, and variances of the investigated variables, as well as in the strength of the bivariate associations between variables [1]. Empirical distinguishability has theoretical significance in that it speaks to whether parents and adolescents need be considered separately in the prediction of HPV vaccination, or if their ratings can be collapsed. Parents and adolescents do agree with regard to some aspects of vaccination, including vaccine self-efficacy [11], but the literature suggests that predictors of intentions to obtain the vaccine differ between parents and adolescents [13]. Therefore, we predict that parents and adolescents will be empirical distinguishable.

After determining whether dyads are empirically distinguishable, researchers utilizing APIM fit the models of interest and assess the existence and size of both actor effects and partner effects [1]. Both effects are interpreted like regression coefficients, characterizing the direction and strength of an association from one predictor to one outcome. Actor effects exist when the association is within person (e.g., parent’s belief that getting the vaccine will be stigmatizing predicting parent’s intent to have their child vaccinated). Partner effects exist when the association is between people (e.g., parent’s belief that getting the vaccine will be stigmatizing predicting the adolescent’s intent to vaccinate). In the literature on HPV vaccination currently, all effects are examined as actor effects. The unique contribution of using APIM in this context is to determine whether any construct known to be important as an actor effect also provides theoretical importance as a partner effect.

Finally, APIM estimates parameters needed to determine whether the pattern of influence is best characterized by an “actor only model” (i.e., an actor effect is nonzero, but the partner effect is zero), a “couple model” (i.e., actor and partner effects are equivalent in size), or a “contrast model” (i.e., actor and partner effects are opposite in direction) [15]. Prior research has almost exclusively approached the study of vaccine uptake as an actor only model, assuming that a parents’ assessment of the risks of HPV influences their own intent to have their daughters vaccinated, but their daughters’ assessments of risk do not influence their intent to have them vaccinated. Possible, but yet untested, is the notion that a mother’s intent to have her daughter vaccinated is predicted equally well by her own assessment of risk as well as her daughter’s assessment of risk (couple model), or a mother’s intent to have her daughter vaccinated is predicted positively by her assessment of the risks of HPV, but negatively by her daughter’s assessments of the risk (contrast model).

### The current study

Due to the limited research on partner effects in the context of HPV vaccination, for the current study, we examined many of the most robust predictors of vaccine acceptance in the literature (i.e., actor effects), looked for the presence of partner effects, and, in an exploratory fashion, allowed the results to inform which models best fit the data. The latter is exploratory only as we are testing known actor effects for the presense of partner effects. It is possible that, had we identified constructs that we hypothesize are partner effects, but have not shown actor effects in the literature, that we would find that different models fit the data better. Nevertheless, there is value in understanding how strong a partner effect is relative to the known actor effect, so we tested the models.

We recruited low-income African American adolescents and their parents to complete a survey regarding HPV vaccination acceptance. Despite nearly identical rates of vaccination for other vaccines (e.g., Tdap, MMR), Black adolescents complete the HPV vaccine course at a lower rate than do other races [2], making this sample particularly important for increasing HPV vaccination rates. Drawing upon prior empirical work, we tested four categories of predictors that were relevant to both the parent and the adolescent: 1) knowledge of HPV and the vaccine, 2) concerns regarding the HPV vaccine, 3) perceived need for an HPV vaccine, and 4) self-efficacy and intent regarding vaccination. These data allowed us to examine four aspects of interdependence, namely:

1. *Are* a*dolescents and parents distinguishable on the basis of their beliefs regarding HPV vaccination*?
2. *Are the actor effects nonzero for all predictors for both adolescents and parents*?
3. *Are the partner effects nonzero for any predictors for either adolescents or parents*?
4. *Which model (i*.*e*., *actor-only*, *couple*, *contrast) best fits each predictor of HPV vaccination*? *(exploratory)*

## Method

### Participants

Participants were 209 dyads of African American adolescents and their parents. Male (*N =* 116) and female adolescents (*N* = 93) were eligible for the study if they self-identified as African American and were between the ages of 11–17 *(M* age = 13.91, *SD* = 1.58 for boys; *M* age = 14.41, *SD* = 1.88 for girls). The adolescents’ parents (*N* = 209; 88% female) were 76% mothers, 9% fathers, 14% guardians [relative], and 1% guardians [non-relative]. Parental/guardian age ranged from 27 to 67 (*M* = 39.66; *SD* = 7.12). Forty-two percent of the parent/guardians were unemployed, and 43% reported working 6–10 hours per week. The modal annual family income was ≤$15,000 (44%; with 33% making $15–30,000, 17% making $30–45,000, and 6% making more than $45,000), and most of parents/guardians had children on the free school lunch program (86%). The majority of the parent/guardians had a high school diploma or GED (35%, with 25% having less than a high school diploma, 29% having attended some college, and 10% having graduated from college).

### Procedures

#### Recruitment

Parent-adolescent dyads were recruited using community outreach and respondent driven sampling between August 2008 and January 2011. *Outreach* involved research staff publicizing the study throughout the community, focusing on census tracks where a high proportion of African Americans reside. Because we enrolled parents of teenage girls and boys, recruitment included canvassing of adolescent-friendly community-based organizations (CBOs) that serve the African American community. These CBOs are located in low-income neighborhoods, and serve residents who are disproportionately vulnerable to STIs such as HPV. Our study team also partnered with the local Housing Authority to recruit adolescents and mothers through a mailing list of public housing apartments that include adolescent children. Finally, research staff recruited participants from an existing wait list of adolescents who expressed an interest in participating in research during the recruitment phase for another recently completed study.

Community outreach was supplemented with *respondent driven sampling* (RDS), where word-of-mouth from past study participants serves to market the study opportunity to other eligible participants. RDS sampling provides an especially helpful method for recruiting populations that can be difficult to reach through traditional strategies, and asks individuals to refer those they know to the project, these individuals in turn refer those they know and so on. RDS procedures consisted of the following. Youth and parent participants who completed the study (“seeds”) were each given two referral cards (four cards total) to distribute to friends and acquaintances who might be interested in study participation. For each qualified youth/mother dyad who was referred to our program and who completed the survey, the "seed” received $5 (up to a maximum of $10 per youth or $10 per mother). Each recruitment card included a participant’s ID number, along with a unique serial number so that we could link recruits to participant recruiters.

#### Parental consent and adolescent assent

Prior to providing any responses, all participants provided written consent (parents) or assent (adolescents), as approved by Syracuse University’s Internal Review Board. Both parents and adolescents were given developmentally appropriate information from the research team regarding the procedures, payment, possible discomforts and risks, benefits, confidentiality, and their right to refuse to participate. After hearing all of the information and having their questions answered, participants provided their written consent/assent and began the assessment.

#### Assessment

Parent-adolescent dyads were recruited using community outreach and respondent driven sampling. After providing written consent (parents) or assent (adolescents), participants completed surveys via audio-assisted computer administered interview (ACASI) at an accessible, storefront, urban research office. Adolescent and parent surveys were completed in separate rooms to optimize privacy. To offset any costs associated with study participation and provide an incentive for participation, each participant (adolescent and parent) was paid $25 to complete the study.

All parents and adolescents began by completing measures regarding descriptive characteristics, HPV and vaccine knowledge, and awareness. Next, because we anticipated that knowledge and awareness of the HPV vaccine would be low, adolescents and their parents’ were provided with an overview of facts concerning HPV vaccination. The overview lasted three minutes and consisted of facts that were presented on the computer screen and read aloud using a digital recording. All participants then completed measures of concerns regarding vaccination and perceived barriers to vaccination, self-efficacy with regard to vaccination, and intentions to vaccinate.

### Measures

#### Descriptive characteristics

To describe the sample, we assessed parent and child age, parent income and employment status, parent education level, and parent race.

**Predictors:** Five categories of predictors were assessed. See Table 1.

**Table 1:** Differences in intent to vaccinate and its predictors as a function of adolescent sex.

	Parents of Girls *n* = 93		Parents of Boys *n* = 116			
	Mean (SD)	%			*t*	*χ*^*2*^
			Mean (SD)	%	*n* = 209	*n* = 209
**Intent to Vaccinate**	4.52 (1.07)		4.68 (1.09)		1.10	
**Knowledge**						
Awareness of the HPV vaccine						
Heard of vaccine?		73%		58%		5.32*
From a TV/radio ad		65%		51%		3.92*
From a healthcare provider		39%		28%		2.91
From a written news article		32%		26%		1.03
From a TV news program		44%		29%		5.52*
From a radio news program		27%		15%		4.81*
From the Internet		13%		13%		0.00
Knowledge about HPV	3.01 (2.38)		2.73 (2.29)		-0.86	
**Concerns**						
Believing getting vaccine will be stigmatizing	2.13 (0.83)		2.25 (0.81)		1.03	
Believing more research is needed on vaccine	2.93 (1.04)		2.77 (0.98)		-0.52	
Believing getting vaccine will lead to greater promiscuity	2.16 (1.03)		2.13 (0.93)		-0.16	
Believing vaccine is unsafe	3.28 (1.10)		3.17 (0.97)		-0.80	
**Perceived Need**						
Believing vaccine will be effective	3.05 (0.68)		3.09 (0.63)		0.45	
Believing HPV is severe	4.59 (0.64)		4.55 (0.69)		-0.37	
Believing one’s child is vulnerable to HPV	3.92 (1.22)		3.82 (1.17)		-0.56	
**Self-Efficacy**	3.12 (0.71)		3.17 (0.69)		0.52	

Independent samples *t*-tests and *χ*^*2*^ tests were conducted with adolescent sex coded 1 = male and 2 = female.

*p < .05.

1. To assess **awareness** of the vaccine, we asked parents and adolescents whether they had heard about the HPV vaccine prior to participation in the study and, if so, how they heard of it. Items were answered with either ‘yes’ (1) or ‘no’ (0) and were analyzed separately.
2. To assess **knowledge** about HPV, possible sequalae, and the vaccine, all parents and adolescents were asked (prior to receiving the informational intervention) nine true or false questions (e.g., “HPV is a sexually transmitted virus”; “HPV can cause cancer”) [16, 17]. Scores were summed to yield a final score (range: 0–9).
3. To assess vaccine-related **concerns**, all parents and adolescents were asked about four types of *concerns* regarding the HPV vaccine: belief that getting the vaccine will be stigmatizing (stigma), belief that more research is needed on the vaccine (research), belief that getting the vaccine will lead to greater promiscuity among the adolescents (promiscuity), and belief that the vaccine is unsafe (safety). Several items were adapted from research conducted by Brewer and colleagues [18] and the remainder developed by our study team. Each concern was assessed with 5 items, except for believing getting the HPV vaccine will lead to greater promiscuity among the adolescents, which was assessed with 6. All items were rated on a scale from 1 (“strongly disagree”) to 6 (“strongly agree”), and scales were created by taking the arithmetic mean for each set of items. Sample items include: “Receiving the HPV vaccine would be embarrassing for my child because it is a vaccine to prevent a sexually transmitted disease” (stigma; α = .72 for parents, α = .81 for adolescents), “There are dangers that we don’t know about in taking the HPV vaccine” (research; α = .78 for parents, α = .65 for adolescents), “The HPV vaccine would encourage my child to be sexually active” (promiscuity; α = .91 for parents, α = .82 for adolescents), and “I would hesitate to get my child vaccinated for HPV because I’ve heard that the vaccine may not be safe” (unsafe; α = .78 for parents, α = .67 for adolescents). All items were also asked of the adolescents, but worded to apply to them rather than “my child.”
4. To assess **perceived need** for HPV vaccination, parents and adolescents completed three scales, each of which was computed by taking the arithmetic mean for each set of items. The first assessed the idea that the vaccine will be *effective* using three items rated on a scale from 1 (“not at all effective”) to 4 (“extremely effective”). Items are: “How effective do you think the HPV vaccine is in preventing genital warts?” “How effective do you think the HPV vaccine is in preventing cervical cancer?” and “How effective do you think the HPV vaccine is in preventing oral cancer?” (α = .81 for parents, α = .64 for adolescents).
5. To assess the *perception that HPV is severe* parents and adolescents completed two items rated from 1 (“not very serious”) to 5 (“extremely serious”) [19]. The items are: “How serious would it be if your child got genital warts caused by HPV?” and “How serious would it be if your child got oral cancer (boys) or cervical cancer (girls) caused by HPV?” The items for the adolescents are identical, except say “you” instead of “your child” (*r* = .85 for parents, *r* = .81 for adolescents).
6. To measure *vulnerability to HPV*, we asked parents and adolescents to endorse their agreement with four statements on a scale from 1 (“strongly disagree”) to 6 (“strongly agree”) [20]. The items include: “If my child did not receive the HPV vaccine, I feel that he or she would be infected with HPV sometime in the future,” and “If my child did not receive the HPV vaccine, I feel that she or he would be very vulnerable to developing cervical cancer or an oral cancer” (α = .91 for parents, α = .91 for adolescents). Again, the items for the adolescents are identical, except say “you” instead of “my child” and “I” instead of “he/she”.
7. To measure **self-efficacy** regarding the vaccine, both parents and adolescents completed a measure containing four items that tapped how easy or difficult it would be for parents to have their adolescent vaccinated (or adolescents to get their parents to have them vaccinated). Items included “How sure are you that you could convince your teen to get the 3 shot HPV vaccination?” and “How sure are you that you could find the time to take your teen for 3 shots of the HPV vaccine?” Each item was rated on a 4-point scale ranging from 1 (“not at all sure”) to 4 (“extremely sure”), and the arithmetic mean of the scale evidenced acceptable reliability (α = .79 for parents, α = .82 for teens).

**Outcome:** We measured **intent to obtain the vaccine** as the outcome of interest in this study. We selected intent because it is the construct most highly correlated with uptake [4, 21], but can vary between the two dyad members. Our measure contained three items, each rated on a 6-point scale from 1 (“very unlikely”) to 6 (“very likely”). For parents of girls and teenage girls themselves, the items were worded concretely (e.g., “Based on what you just learned, how likely are you to get your daughter vaccinated with the HPV vaccine?”), whereas for parents of sons and the teenage boys themselves, the items were hypothetical (e.g., “Based on what you just learned, how likely would you be to get your son vaccinated with the HPV vaccine if it were approved for use with boys?”). This was done because at the time of data collection, the vaccine was not available for boys. Reliability for both was acceptable (α = .81 for parents with sons, α = .79 for parents with daughters, α = .69 for teenage girls, α = .60 for teenage boys).

## Results

### Preliminary analyses

None of the descriptive variables (e.g., income, age of the adolescent, and whether the adolescent had prior sexual intercourse) were associated with the outcomes of interest. Controlling for these variables did not change any of the associations; therefore, the results described are those conducted without any covariates. Also, there were no differences in the bivariate associations among the study variables and intent to vaccinate as a result of adolescent sex (see Table 1). Therefore, we conducted the main analyses with data from the two sexes combined.

### Main analyses

First, we examined whether adolescents and parents differed with respect to their beliefs regarding HPV vaccination. Results indicate that there is statistical advantage to treating parents and adolescents as distinguishable. Specifically, models in which the intercepts, actor effects, partner effects, and error variances are fixed to be equivalent across parents and adolescents results in poor model fit for all variables examined (all *p*s < .001; see the first column of results in Table 2).

**Table 2:** Results from Actor-Partner Interdependence Model (APIM) analyses.

			DV: Intent to Vaccinate																	
			Actor Effects									Partner Effects								
			Parent				Adolescent					Parent					Adolescent			
	*χ*^*2*^		b	(se)	*t*			b	(se)	*t*		b	(se)	*t*		b		(se)	*t*	
**Knowledge**																				
Heard of Gardasil?	**52.33**	***	-.15	(.16)	-.96			.39	(.16)	**2.39**	*	-.06	(.17)	-.37		-.06		(.15)	-.42	
Knowledge about HPV	**46.96**	***	.02	(.03)	.63			.14	(.05)	**2.63**	**	-.01	(.03)	-.42		-.05		(.05)	-1.10	
**Concerns**																				
Believing getting Gardasil will be stigmatizing	**36.04**	***	-.21	(.09)	**-2.26**	*		-.32	(.07)	**-4.65**	***	-.00	(.10)	-.02		.08		(.07)	1.21	
Believing more research is needed on Gardasil	**63.32**	***	-.39	(.07)	**-5.43**	***		-.32	(.10)	**-3.14**	**	.05	(.08)	.65		-.02		(.09)	-.21	
Believing getting Gardasil will lead to greater promiscuity	**58.15**	***	-.18	(.08)	**-2.36**	*		-.19	(.08)	**-2.29**	*	-.03	(.08)	-.31		.09		(.08)	1.15	
Believing Gardasil is unsafe	**72.38**	***	-.44	(.07)	**-6.59**	***		-.09	(.09)	-1.08		.12	(.08)	1.45		.06		(.07)	.88	
**Perceived Need**																				
Believing Gardasil will be effective	**44.03**	***	.51	(.11)	**4.63**	***		.63	(.12)	**5.20**	***	-.07	(.12)	-.56		.02		(.11)	.16	
Believing HPV is severe	**59.05**	***	.06	(.11)	.51			.34	(.11)	**3.01**	**	-.00	(.12)	-.01		.04		(.11)	.35	
Believing one’s child is vulnerable to HPV	**52.91**	***	.39	(.06)	**6.92**	***		.27	(.06)	**4.88**	***	-.05	(.06)	-.77		.01		(.05)	.29	
**Self-Efficacy**	**21.02**	***	.44	(.10)	**4.21**	***		.55	(.09)	**5.88**	***	-.24	(.11)	**-2.25**	*	-.11		(.09)	-1.19	

Actor-Partner Interdependence Model results. *χ*^*2*^ value represents a test of whether the dyad members are distinguishable on the given construct in terms of intercept, actor effect, partner effect, or error variances. A significant *χ*^*2*^ value indicates the members are distinguishable. All remaining values are unstandardized solutions from two-intercept models utilizing grand mean centered predictors. Actor effect refers to the effect of that predictor (row) on intent to vaccinate for the same person. Partner effect refers to the effect of that predictor on intent to vaccinate for the other person.

***p < .001

**p < .01

*p < .05

Second, we examined which actor effects are significant among parents and adolescents. Each predictor was tested independently in a two-intercept model with intent to vaccinate as the outcome that contained: (a) a dummy code for parent (coded 0 = adolescent; 1 = parent), (b) an interaction between the dummy code and the actor effect of the predictor, and (c) an interaction between the dummy code and the partner effect of the predictor. Complete results from these analyses can be seen in Table 2 under the heading “actor effects.” Briefly, for parents, all actor effects were significantly different from zero, except for the constructs tapping knowledge of HPV and whether HPV is severe. For adolescents, all actor effects were significantly different from zero except for the belief that the vaccine is unsafe.

Third, we examined which partner effects were significant among parents and adolescents utilizing the same models as tested for the actor effects. Complete results from these analyses can be seen in Table 2 under the heading “partner effects.” Briefly, only one partner effect was significantly different from zero, and that was the effect of parent’s self-efficacy on their adolescent’s intent, which predicts in the opposite direction from the actor effect (i.e., as parents self-efficacy increases, their adolescent’s intent decreases).

Fourth, we examined which model (i.e., actor-only, couple, contrast) best fits each predictor of HPV vaccination for each dyad member. To determine which model was best fitting, we followed a procedure described by Kenny and Ledermann [15], in which we first calculated *k*, or the ratio of the partner effect to the actor effect. Next, we used the Monte Carlo method to construct 95% confidence intervals around this parameter. Finally, we assigned a model depending on whether the confidence interval included -1 (contrast model), 0 (actor-only model), or 1 (couple model). In general, the actor-only model fit most of our predictors, with a few exceptions (see Table 3). Notably, a contrast model was plausible for parents when looking at the predictors of believing the vaccine is stigmatizing and believing the vaccine will lead to greater promiscuity. A contrast model was also plausible for adolescents when considering self-efficacy. Finally, a couple model was plausible for adolescents when considering beliefs that HPV is severe. These are described in greater detail subsequently, in the discussion section.

**Table 3:** Relative size of actor and partner effects *(k*).

	Parent			Adolescent		
	*k*	CI of *k*	Model	*k*	CI of *k*	Model
**Knowledge**						
Heard of Gardasil?	.42	-8.79, 9.16	n/a	-.16	-1.70, 1.08	n/a
Knowledge about HPV	-2.57	-23.97, 21.67	n/a	-.11	-.84, .65	n/a
**Concerns**						
Believing getting Gardasil will be stigmatizing	-.38	-1.92, .43	contrast, actor-only	.01	-.60, .73	actor-only
Believing more research is needed on Gardasil	.05	-.42, .54	actor-only	-.17	-.91, .41	actor-only
Believing getting Gardasil will lead to greater promiscuity	-.48	-2.64, .49	contrast, actor only	.14	-1.16, 1.95	n/a
Believing Gardasil is unsafe	-.15	-.48, .19	actor-only	-1.23	-11.63, 10.33	n/a
**Perceived Need**						
Believing Gardasil will be effective	.04	-.46, .51	actor-only	-.10	-.53, .26	actor-only
Believing HPV is severe	.64	-10.80, 11.23	n/a	-.003	-.83, 1.01	couple, actor-only
Believing one’s child is vulnerable to HPV	.04	-.21, .30	actor-only	-.18	-.72, .30	actor-only
**Self-Efficacy**	-.25	-.77, .17	actor-only	-.44	-.91, -.06	contrast, actor-only

The k statistic is a ratio of the size of the partner effect to the actor effect. The Confidence Interval of k is a 95% confidence interval using the Monte Carlo Method. Models are assigned using the following rule: if k = 0 then actor-only, if k = -1 then contrast, if k = 0 then couple. n/a is presented as a model when the CI is too wide to estimate which model is best (i.e., all three models are plausible).

### Supplemental analyses

Because of the robust support for actor-only models, in which each actors’ predictors contributed most strongly to their own level of intention, we close by providing information relevant to understanding which of the predictors exert the greatest influence on the outcomes when controlling for the effects of all others. Because past research suggests parents make HPV vaccination decisions for their adolescents, we identified the best predictors of parental intent. First, we entered all predictors that were significantly associated parents’ intent in bivariate tests into a simultaneous multiple regression model to examine the relative contribution of each to the amount of variance in intent explained. In this model, believing the vaccine is unsafe (*β =* -.28, *t*(201) = -3.03, *p* < .01) and perceiving one’s child is vulnerable to HPV (*β =* .30, *t*(201) = 4.36, *p* < .001) remained significant predictors above and beyond the others (see Table 4 for complete results).

**Table 4:** Predicting self-efficacy and intent to vaccinate among parents.

	Predicting Self-Efficacy			Predicting Intent to Vaccinate		
	OLS Regression		Relative Weight	OLS Regression		Relative Weight
	β	t		β	t	
Awareness						
Heard of vaccine?						
Knowledge about HPV	.11	1.89	5.38			
Concerns						
Believing getting vaccine will be stigmatizing	-.20	-2.24*	16.91*	.10	1.04	1.76
Believing more research is needed on vaccine	-.10	-1.14	12.49*	-.02	-0.23	12.48*
Believing getting vaccine will lead to greater promiscuity	-.004	-0.05	8.28*	-.12	-1.38	3.83
Believing vaccine is unsafe	-.17	-2.07*	15.98*	-.28	-3.03**	26.83*
Perceived Need						
Believing vaccine will be effective	.02	0.30	3.48	.09	1.22	12.27
Believing HPV is severe	.18	3.02**	12.84*			
Believing one’s child is vulnerable to HPV	.12	1.85	8.09*	.30	4.36***	39.18*
Barriers						
Access to healthcare	.20	3.20**	12.42*			
Trust in physicians				.05	0.75	3.64
Fearing discrimination in healthcare						
Having experienced discrimination in healthcare	-.05	-0.78	4.13			

OLS regression results obtained in a simultaneous multiple regression model. Relative weights presented are rescaled weights (adding up to 100%), with asterisks indicating that the alpha < .05 confidence intervals around the weight, based on 10,000 bootstrapped resamples, does not include zero.

***p < .001

**p < .01

*p < .05.

Our predictors were correlated, however, and as such we did not wish to rely on these results exclusively. We supplemented them with relative weight analysis (RWA), which is a procedure for estimating the relative importance of correlated predictors in a regression equation [22, 23]. This technique uses a variable transformation approach that creates a new set of predictors that are orthogonal to each other, then regresses the outcome onto these orthogonal predictors, allowing us to interpret the resulting standardized regression coefficients without multicollinearity having negative effects. When we entered all of the predictors that were significant in the multiple regression model described previously, the results corroborated what was found with multiple regression: the most influential predictors of intent were believing one’s child is vulnerable to HPV (39% of the explained variance) and believing the HPV vaccine is unsafe (27%).

## Discussion

Despite the promise of the HPV vaccine, public acceptance remains low as compared to other vaccines in the US [2]. In this study, by taking a dyadic perspective, we sought to better understand the predictors of vaccine intent among a sample of low-income African American male and female adolescents and their parents. We obtained several findings that warrant discussion.

The decision to obtain the HPV vaccine is unique from many health-promoting decisions in that it involves a dyad. Because the recommended age of vaccination is 11–12 years old, parents are primarily responsible for obtaining the vaccine for their children [8], but we hypothesized that bidirectional influence would exist with regard to HPV vaccination such that adolescents and parents influence each other [13, 11]. There was some evidence in favor of this hypothesis. First, we found that parents and adolescents are empirically distinguishable, meaning that the patterns of associations among variables related to HPV vaccination are not uniform across the two groups [1]. The practical implication of this is that interventions need to be targeted specifically at each member of the dyad; processes that underlie HPV vaccination decisions are not the same for parents as they are for their adolescents.

Next, we examined whether adolescents’ and parents’ beliefs regarding HPV vaccination influence each other’s intent to obtain the vaccine, by examining whether several known actor effects in the literature also produce partner effects. Results comparing relative sizes of actor and partner effects suggest a few beliefs do. First, we found evidence for a couple model with regard to HPV severity. Couple models exist when actor and partner effects are equivalent, and thus both individuals equally contribute to the outcome [15]. In our data, the more a parent believed HPV was severe, the more the adolescent intended to obtain the vaccine.

We found more evidence for contrast models. A contrast model is one in which the actor effect and partner effect are equal in size, but in opposite directions [15]. In our data, we found three notable contrast models. The first two deal with parent’s intent to vaccinate: when adolescents believe the vaccine will lead to greater promiscuity or be stigmatizing to obtain, their parents were more likely to intend to obtain the vaccine for them. Both of these beliefs are negatively associated with parents’ intent when held by the parent themselves, however. The third deals with adolescent’s intent to vaccinate; parent’s self-efficacy is negatively associated with an adolescent’s intent to vaccinate, despite an adolescent’s own self-efficacy being positively associated with their intent.

Other than these models, however, most predictors were found to be actor-only models, in which the actor effect is non-zero, but the partner effect is zero. This is the model assumed by the literature; that is, the majority of the HPV literature has examined individual-level data with regard to HPV vaccination, and our results suggest that in most cases this is empirically reasonable. Practically, this suggests that, with the exceptions noted previously, interventions aimed at changing the beliefs of one member of the dyad will impact that member’s intent to vaccinate, with little impact on the other member of the dyad. However, it is important to remember that these results were obtained by examining known actor effects to see if partner effects emerged as well. Another avenue of intervention development that should be considered is to specifically test predictors that are hypothesized to be important between individuals (e.g., adolescent’s interest in sexual activity may not influence the adolescent him or her self, but may influence the parent as a partner effect).

### Strengths, limitations, and future directions

To our knowledge, this work is the first HPV vaccine research to sample (a) low-income African American adolescents, (b) including males as well as females, and (c) their parents (d) using dyadic methods. Sampling both female and male adolescents, as well as their parents, provided us a unique opportunity to investigate how vaccination uptake manifests in this vulnerable population sub-group. Further, using sophisticated statistical techniques, we were able to take advantage of the richness of these data. This work is not without limitations, however. Due to the cross-sectional nature of these data, we cannot speak to mechanisms or causal ordering of the effects. Future research is needed to untangle why contrast models are plausible for several predictors of vaccine intent, as these could have implications for designing interventions aimed at increasing acceptance. For example, one of our obtained contrast models suggests that bolstering a parent’s self-efficacy is likely to lower their adolescent’s intent to obtain the vaccine. This could be problematic if both members of the dyad have input into the final decision to obtain the vaccine. Additionally, we did not explicitly ask participants how much they anticipated their attitude or behavioral intentions would be influenced by their partner (e.g., we did not ask parents how much they would allow their adolescent’s attitudes influence them). Our models approach this topic by looking for statistical influence, but future research would benefit from explicitly asking the actors how much their partner influences them in this domain.

Despite these limitations, there is an important clinical and public health implication of this work that should be emphasized. Because most predictors were well-fitted by actor-only models, interventionists can be confident that the extant literature, in which predictors of vaccine intent and uptake are examined within individuals, holds well even in dyadic contexts. Thus, interventions targeted at individuals based on this literature are likely to be successful. For marketers and interventionists alike, our results suggest that parents must believe their child is vulnerable to HPV prior to intending to obtain it. This suggests that education aimed at publicizing the prevalence of HPV would help with vaccine uptake, as would informing both parents and their adolescents about what causes vulnerability to HPV. We saw little evidence that adolescent attitudes influence parents’ self-efficacy and intent; however, if an adolescent knows that a particular part of their lifestyle makes them vulnerable to HPV, and they are aware of the risks of HPV, they may communicate their vulnerability to their parent, thus enhancing uptake. It may also be beneficial to normalize the risk of HPV from a marketing and intervention standpoint. By virtue of their age, all sampled adolescents were at some risk for HPV, or would be, yet parents were not overwhelmingly aware of their own adolescents’ vulnerability, which undermined their vaccine uptake intentions. Instead of relying on adolescents to communicate their lifestyle details to their parents, marketing and interventions aimed at generating the idea that all adolescents are vulnerable to HPV could enhance uptake.

### Conclusions

HPV vaccine uptake has not continued to increase at the rates expected [2], suggesting a fresh approach to understanding the causal factors behind vaccine acceptance is needed. In the current study, we employed dyadic statistical methods to determine whether considering parent-adolescent dynamics could provide additional explanatory power. For some dimensions of HPV vaccination beliefs, we found evidence that the dyadic context could be very influential and, with additional research, has the potential to bolster HPV vaccination uptake.
